# Decreasing prevalence of transmitted drug resistance among ART-naive HIV-1-infected patients in Iceland, 1996–2012

**DOI:** 10.1080/20008686.2017.1328964

**Published:** 2017-06-13

**Authors:** Malik Sallam, Gülşen Özkaya Şahin, Hlynur Indriðason, Joakim Esbjörnsson, Arthur Löve, Anders Widell, Magnus Gottfreðsson, Patrik Medstrand

**Affiliations:** ^a^ Faculty of Medicine, Department of Translational Medicine, Lund University, Malmö, Sweden; ^b^ Clinical Microbiology, Laboratory Medicine Skåne, Lund, Sweden; ^c^ Faculty of Medicine, Department of Laboratory Medicine, Section of Microbiology, Immunology and Glycobiology, Lund University, Lund, Sweden; ^d^ Faculty of Medicine, School of Health Sciences, University of Iceland, Reykjavik, Iceland; ^e^ Nuffield Department of Medicine, University of Oxford, Oxford, UK; ^f^ Microbiology, Tumor and Cell Biology, Karolinska Institute, Stockholm, Sweden; ^g^ Department of Virology, Landspítali University Hospital, Reykjavik, Iceland; ^h^ Department of Infectious Diseases, Landspítali University Hospital, Reykjavik, Iceland

**Keywords:** Phylogeny, resistance, trend, transmission, BEAST

## Abstract

**Introduction:** Resistance to antiretroviral drugs can complicate the management of HIV-1 infection and impair control of its spread. The aim of the current study was to investigate the prevalence and transmission of HIV-1 drug resistance among 106 antiretroviral therapy (ART)-naïve patients diagnosed in Iceland (1996–2012).

**Methods:** HIV-1 polymerase sequences were analysed using the Calibrated Population Resistance tool. Domestic spread of transmitted drug resistance (TDR) was investigated through maximum likelihood and Bayesian approaches.

**Results:** Among ART-naïve patients, the prevalence of TDR to any of the following classes (NRTIs, NNRTIs and PIs) was 8.5% (95% CI: 4.5%- 15.4%): 6.6% to NRTIs, 0.9% to NNRTIs, and 1.9% to PIs. The most frequent NRTI mutation detected was T215C/D (n=7, 5.7%). The only NNRTI mutation detected was K103N (n=1, 0.9%). PI mutations detected were M46I (n=1, 0.9%) and L90M (n=1, 0.9%). Six patients harbouring T215C/D, were linked in a supported phylogenetic cluster. No significant association was found between TDR and demographic or risk groups. Trend analysis showed a decrease in the prevalence of TDR (1996–2012, p=0.003).

**Conclusions:** TDR prevalence in Iceland was at a moderate level and decreased during 1996-2012. Screening for TDR is recommended to limit its local spread and to optimize HIV-1 therapy.

**A** **bbreviations**: ART: Anti-retroviral therapy; ARV: antiretroviral; ATV/r: atazanavir/ritonavir; AZT: azidothymidine; BEAST: Bayesian evolutionary analysis by sampling trees; CI: confidence interval; CPR: calibrated population resistance; CRF: circulating recombinant form; d4T: stavudine; EFV: efavirenz; FET: Fishers’ exact test; FPV/r: fosamprenavir/ritonavir; HET: heterosexual; IDU: injection drug use; IDV/r: indinavir/ritonavir; LPV/r: lopinavir/ritonavir; MSM: men who have sex with men; M-W: Mann–Whitney *U* test; NFV: nelfinavir; NNRTIs: non-nucleoside reverse transcriptase inhibitors; NRTIs: nucleoside reverse transcriptase inhibitors; NVP: nevirapine; PIs: protease inhibitors; *pol*: polymerase gene; SDRM: surveillance drug resistance mutation; SQV/r: saquinavir/ritonavir; TDR: transmitted drug resistance

## Introduction

HIV-1 was first reported in Iceland in 1985. By the end of 2012 a total of 300 patients had been diagnosed with HIV-1 infection in the country, of which 66 had developed AIDS and 39 passed away as a result of the disease.[1,2] Following the first introduction of HIV-1 to Iceland onwards to the end of 2012, the infection has been dominated by clade B with a relatively low fraction of founders compared to the total number of introductions.[3] HIV-1 infection in the country appeared to be highly concentrated among men who have sex with men (MSM) and injection drug users (IDUs) and less among heterosexuals (HET). The genetic diversity of HIV-1 in Iceland has increased significantly over time, most likely related to the increased proportion of foreign-born residents in the country from the mid-1990s.[3,4]

Successful management of HIV-1 infected patients is highly dependent on antiretroviral therapy (ART) that has been shown to reduce morbidity and mortality from the disease.[5] ART also contributes to the prevention of HIV-1 spread, as lowering the viral load diminishes the risk of HIV-1 transmission.[6–8]

Antiretroviral (ARV) drug resistance mutation can arise as a result of direct transmission from a patient harbouring drug-resistant mutants (primary or transmitted drug resistance, TDR) or due to selection of drug-resistant mutants within patients who are not successfully managed (secondary or acquired resistance). The rapid evolution of HIV-1 facilitated by its error-prone reverse transcriptase and selection pressure by the ARV drugs are the driving forces behind the emergence of ARV drug resistance.[9,10] Resistant strains can be transmitted, causing early treatment failures in the newly infected individuals. Thus, TDR jeopardizes both preventive and treatment efforts in patients.[11] Acquired resistance can emerge when the treatment regimens fail to suppress the viral load, most commonly due to suboptimal adherence of the patients. Patients with ARV resistant mutants are at high risk of treatment failure with risk of forward transmission of these mutant variants.[12–14] Therefore, WHO highly recommends testing patients who are newly diagnosed with HIV-1 infection for ARV drug resistance.[15]

No earlier studies have investigated the status of ARV drug resistance in Iceland. The objectives of the current study were to conduct an epidemiological surveillance on TDR among patients who were diagnosed with HIV-1 infection in Iceland between 1996 and 2012 and to describe the types of resistance to different classes of ARV drugs in the country, as well as to investigate the domestic transmission of TDR.

## Materials and methods

### Study population

Iceland is a Nordic country with a population of about 320,000 people as of the end of 2012.[2] The study comprised 106 HIV-1 infected patients diagnosed in Iceland during 1996–2012. Limited availability of concurrent plasma samples from patients diagnosed prior to 1996 precluded the inclusion of such patients in the study. Patient data included age, sex, self-reported risk factor, country of birth and self-reported country of infection. HIV-1 subtype/circulating recombinant form (CRF) data were obtained from a previous work that included our study population.[3] The inclusion criteria were as follows: (1) patients newly diagnosed with HIV-1 infection; (2) plasma samples drawn within six months of diagnosis. The exclusion criteria were as follows: (1) known exposure to ART based on medical records or self-reporting; (2) previous diagnosis of HIV-1 infection outside Iceland.

### Ethical approval

The study was approved by Landspitali University Bioethics Committee in accordance with the declaration of Helsinki. Since many patients at the time of our study initiation were deceased or had moved outside Iceland, the Landspitali University Hospital Ethics Committee considered the use of the study material for the research in this study without the need of consent of the study participants since it would be impossible or impractical to obtain.

### Genotypic HIV-1 resistance testing

The HIV-1 polymerase (*pol*) sequences that were utilized in our study were generated using the Sanger population sequencing method. The details of the sequencing approach were described previously.[3] The 106 partial *pol* sequences (1020 base pairs; nucleotide positions 2268–3287 of HXB2, GenBank accession number K03455) were analysed using the calibrated population resistance (CPR) tool in the Stanford University HIV drug resistance database (http://cpr.stanford.edu/cpr.cgi).[16] The 2009 Stanford Surveillance Drug Resistance Mutation list include the following codon sites in the protease region: 23, 24, 30, 32, 46, 47, 48, 50, 53, 54, 73, 76, 82, 83, 84, 85, 88, 90, and the following codon sites in the reverse transcriptase region: 41, 65, 67, 69, 70, 74, 75, 77, 100, 101, 103, 106, 115, 116, 151, 179, 181, 184, 188, 190, 210, 215, 219, 225, 230.[16]

### Maximum likelihood phylogenetic analysis

A maximum likelihood (ML) phylogenetic tree was constructed for the Icelandic subtype B sequences (*n *= 63) in GARLI v2.0 using the GTR+I + Γ nucleotide substitution model.[17] Statistical support was determined using approximate likelihood ratio test Shimodaira–Hasegawa (aLRT SH-like) in PhyML v3.1, and aLRT-SH values of more than or equal to 0.9 were considered significant.[18,19] Five runs in GARLI were conducted and the tree with the highest likelihood value was retained for analysis. The ML analysis was repeated after removal of resistance codon positions in the protease region (30, 32, 46, 47, 48, 50, 54, 82, 84, 88 and 90) and in the reverse transcriptase region (41, 62, 65, 67, 70, 74, 75, 77, 100, 101, 103, 106, 108, 115, 116, 151, 181, 184, 188, 190, 210, 215, 219, 225, 227 and 230) to reduce potential bias due to ARV selective pressure.[20] Following removal of aforementioned sites, the sequence alignment had a length of 909 base pairs.

### Bayesian phylogenetic analysis

The Icelandic clade B sequences were analysed using a Bayesian approach in BEAST v1.8.2.[21] Analysis was done using a constant coalescent population model with relaxed lognormal uncorrelated molecular clock with an uninformative rate prior and HKY85 nucleotide substitution model (Supplementary File 1). Five runs, each of 100 million steps in the Markov chain, were performed using BEAST and then combined using LogCombiner v1.8.2 in BEAST package.[21] Convergence was checked using Tracer v1.6.0 (http://beast.bio.ed.ac.uk/Tracer), and BEAST parameters showed high convergence with effective sample size (ESS) values ≥ 200 for all. The maximum clade credibility (MCC) tree was generated using TreeAnnotator v1.8.0 available in BEAST package.[21] For branch support, posterior probability (*PP*) values equal to 1.0 were considered significant. The analysis was repeated after removal of resistance codon positions as described previously.

### Statistical analysis

The binomial distribution (Wilson score interval) 95% confidence interval of the prevalence was calculated using EpiTools epidemiological calculator available online (http://epitools.ausvet.com.au). P-values were calculated using the exact two-sided Fisher’s test (FET) through GraphPad Software, Inc., available online (http://graphpad.com/quickcalcs/contingency1/). Mann–Whitney *U* test (M-W) was done using the VassarStats website, available online (http://vassarstats.net/utest.html). For trend analysis, we used two-tailed linear-by-linear test for association through IBM SPSS Statistics 21.0.

### Sequence accession numbers

We have selected 30 partial *pol* sequences included in this study to be deposited in GenBank. These sequences were assigned with the following accession numbers: KY084400–KY084429.

## Results

### Characteristics of the study population

Out of 209 patients who were diagnosed with HIV-1 infection in Iceland during 1996–2012, 106 had samples which met the inclusion criteria of the study and were included for subsequent analysis. The characteristics of the study subjects are illustrated in Table 1.

**Table 1.** Characteristics of the HIV-1 infected individuals included in the study.

	All	
Characteristic	*n*^a^	%
**Total**	106	
**Sex**		
*Male*	64	60
*Female*	42	40
**Riskfactor^b^**		
*MSM*	29	27
*HET*	45	42
*IDU*	26	25
*MTCT*	1	1
*Unknown*	5	5
**Country of birth**		
*Iceland*	65	61
*Non-Iceland*	41	39
**Country of infection^c^**		
*Iceland*	39	37
*Non-Iceland*	51	48
*Unknown*	16	15

^a^ *n*: number; ^b^Risk factor: Self-reported risk factor for HIV-1 acquisition (MSM: men who have sex with men; HET: heterosexual; IDU: injection drug use; MTCT: mother to child transmission; ^c^Country of infection: self-reported country of infection.

Approximately 60% of the patients were males and 40% were females. The median age at the time of diagnosis was 34 years (range: 2–77 years). Males were found to be significantly older than females (median age 37 vs. 30 years, respectively, *p *< 0.001, M-W). The median time between diagnosis and sampling was seven days (range: 0–154 days).

Self-reported routes of HIV-1 infection were: HET (*n *= 45; 42%), MSM (*n *= 29; 27%) and IDU (*n *= 26; 25%). A high proportion of the patients (*n *= 65; 61%) were born in Iceland, and approximately 50% of all patients reported to have been infected outside the country.

The distribution of the study participants in terms of sex and risk factor for HIV-1 acquisition was nearly matched with the total number of diagnosed patients during the same period. The exception was the (unknown/others) risk factor category, which was under-represented in our sample (Table 2). To exclude selection bias in samples with regard to year of diagnosis, we compared the total number of individuals who were diagnosed with HIV-1 infection to the number of samples that were analysed by dividing the study period into two time intervals, 1996–2004 and 2005–2012. Similar proportions of sampling were found for the intervals (50.6% vs. 50.7%).

**Table 2.** Distribution of the study subjects compared to the total population of patients who were diagnosed during the same time interval.

	Study subjects		Total population		Coverage
Characteristic	*n*^a^	%	*n*	%	%
**Total**	106		209		51
**Sex**					
*Male*	64	60	134	64	48
*Female*	42	40	75	36	56
**Risk Factor^b^**					
*MSM*	29	27	48	23	60
*HET*	45	42	81	39	56
*IDU*	26	25	45	22	58
*Others/unknown*	6	6	35	17	17

^a^ *n*: number; ^b^Risk factor: self-reported risk factor for HIV-1 acquisition (MSM: men who have sex with men; HET: heterosexual; IDU: injection drug use; Others: mother to child transmission, blood transfusion.

### Distribution of HIV-1 subtypes/CRFs

Subtype B was found to be the most common genetic variant in the study sample (*n *= 63; 59%) followed by CRF01_AE (*n *= 13; 12%), subtype C (*n *= 10; 9%), CRF02_AG (*n *= 8; 8%) and sub-subtype A1 (*n *= 6; 6%). The minor subtypes/CRFs included: subtype D (*n *= 2), CRF14_BG (*n *= 1), CRF16_A2D (*n *= 1), CRF40_BF (*n *= 1) and CRF45_cpx (*n *= 1). For subsequent analysis related to subtypes/CRFs, we have divided the samples into subtype B (*n *= 63) and non-B subtypes/CRFs (*n *= 43) groups.

### Transmitted drug resistance (TDR)

The prevalence of ARV drug resistance among the study population to any of the following classes: nucleoside reverse transcriptase inhibitor (NRTI), non-nucleoside reverse transcriptase inhibitor (NNRTI) and protease inhibitor (PI), was found to be 8.5% (95% CI: 4.5%–15.4%). In the nine patients that harboured ARV drug resistance mutations, seven had at least one mutation suggestive of resistance to NRTIs, two patients had at least one mutation indicative of resistance to PIs and one patient had a mutation indicative of resistance to NNRTIs. A single patient had mutations suggestive of resistance to both NRTIs and PIs. None of the patients had a sequence with mutations to all three classes of ARV drugs.

Thymidine analogue 215 revertant mutants (T215C/D), that cause low-level resistance to azidothymidine (AZT) and stavudine (d4T), dominated the NRTI mutations detected (*n *= 7; 6%). The other NRTI mutation detected was M41L (*n *= 1; 0.9%), which causes intermediate resistance to AZT and d4T. The only major NNRTI mutation detected was K103N (*n *= 1; 0.9%), which causes high-level resistance to efavirenz (EFV) and nevirapine (NVP). The two major PI mutations found were M46I (*n *= 1; 0.9%), which causes low-level resistance to nelfinavir (NFV) and L90M (*n *= 1; 0.9%), which causes high-level resistance to NFV, intermediate resistance to indinavir/ritonavir (IDV/r) and saquinavir/ritonavir (SQV/r) and low-level resistance to atazanavir/ritonavir (ATV/r), fosamprenavir/ritonavir (FPV/r) and lopinavir/ritonavir (LPV/r).[16]

The prevalence of TDR was higher in patients who were born in Iceland (12.3% vs. 2.5%), and in patients who reported infection in Iceland (10.2% vs. 2%); however, the difference was not statistically significant. Stratified by risk factor, TDR was found to have a higher prevalence in HET (11.1%) and MSM (10.3%) in comparison to IDU (3.8%) but without statistical significance. TDR was also more prevalent among patients infected with subtype B compared to all non-B subtypes/CRFs (12.7% vs. 2.3%) but also without statistical significance (Table 3).

**Table 3.** Characteristics of ART-naive patients with and without TDR.

	TDR^a^		Non-TDR		*p*-value
	*n*^b^	%	*n*	%	TDR vs. non-TDR
**Patients no.**	9		97		
**Sex**					
*Male*	5	56	59	61	1.00
*Female*	4	44	38	39	
**Risk factor**					
*MSM ^c^*	3	33	26	27	MSM vs. HET: 1.00
*HET ^d^*	5	56	40	41	HET vs. IDU: 0.40
*IDU ^e^*	1	11	25	26	MSM vs. IDU: 0.61
*MTCT ^f^*	0	0	1	1	
*Unknown*	0	0	5	5	
**Country of birth**					
*Iceland*	8	89	57	59	0.15
*Non-Iceland*	1	11	40	41	
**Reported country of Infection**					
*Iceland*	6	50	37	34	0.16
*Non-Iceland*	2	17	59	54	
*Unknown*	4	33	14	13	
**Subtype**					
*B*	8	89	55	57	0.08
*Non-B*	1	11	42	43	

^a^TDR: transmitted drug resistance; ^b^ *n*: number; ^c^MSM: men who have sex with men; ^d^HET: heterosexual; ^e^IDU: injection drug use; ^f^MTCT: mother-to-child-transmission.

**Figure 1.**
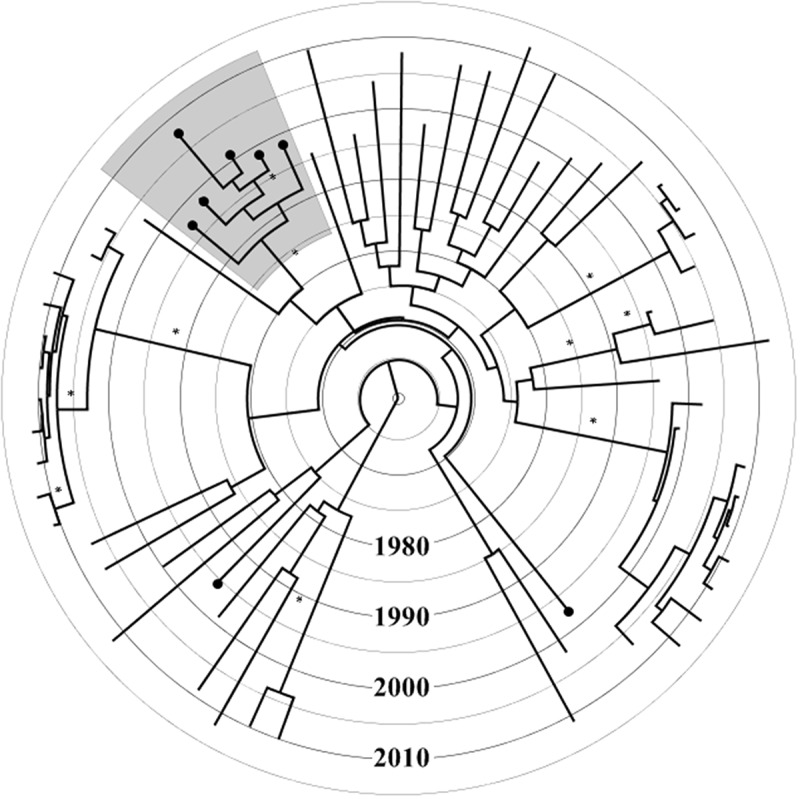
Time-resolved maximum clade credibility tree of 63 subtype B Icelandic sequences. The tree was constructed using TreeAnnotator v1.8.0 included in BEAST software package. Branches with posterior probability value of 1.0 are marked with an asterisk. Terminal branches marked with a black circle at the tip represent sequences harbouring at least one transmitted drug resistance (TDR) mutation. The grey shaded area represents the monophyletic cluster with TDR mutation (T215C/D).

Maximum likelihood analysis revealed that six out of eight subtype B sequences, which harboured TDR mutations, were part of a single phylogenetic cluster with high statistical support (aLRT-SH like value of 1.0). The patients within this cluster harboured T215C/D and were diagnosed between 1997 and 2008. Three patients were males, two of whom reported MSM and one reported HET as risk factors for HIV acquisition. Three patients were females who reported HET as a risk factor for HIV acquisition. An identical cluster was found in the ML tree constructed after removal of resistance mutation codon sites. This excludes the possibility of incorrect clustering due to convergent evolution. The same transmission cluster was also identified through Bayesian analysis before and after removal of resistance mutation codon sites, with a *PP* value of 1.0 in the MCC tree (Figure 1). The time to the most recent common ancestor of the cluster harbouring (T215C/D) dated back to 1989 (median estimate, 95% highest posterior density interval: 1983–1994).

### Temporal trend of TDR

When we compared patients diagnosed before mid-2004 (*n *= 40) to those with dates of diagnosis after mid-2004 (*n *= 66), we found a significant higher likelihood of harbouring drug resistance mutations in the earlier time interval (*p *= 0.025, FET).

**Figure 2.**
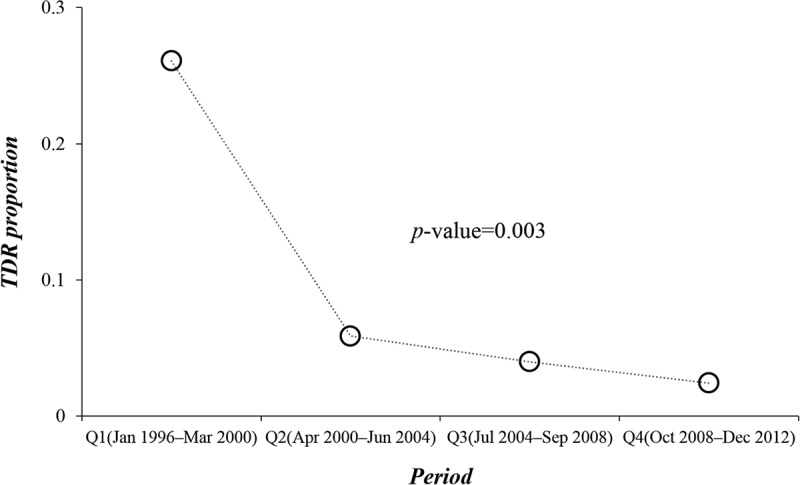
Temporal trend of transmitted drug resistance (TDR) over the study period. The horizontal axis represents the study period divided into four quarters, each of which represents 4.25 years. The vertical axis represents proportion of TDR in each quarter. The *p*-value indicates the result of trend analysis conducted using linear-by-linear test for association.

To investigate the trend of TDR prevalence and subtype distribution over time, we divided the study period into quarters, each of which represented 4.25 years. The proportion of patients with TDR was 26.0, 5.9, 4.0 and 2.4% for the four quarters, respectively. A significant decline of TDR prevalence was noted during 1996–2012 (*p *= 0.003; LBL, Figure 2). However, the decrease of TDR did not display a significant change over the latter three quarters (April 2000–December 2012, *p *= 0.518; LBL). No statistical difference was found upon tracking the temporal changes in subtype B vs. non-B subtypes/CRFs over the study period (*p *= 0.493; LBL).

## Discussion

In the current study, we assessed the prevalence of TDR among HIV-1 infected, ART-naive patients in Iceland over a period of 17 years. By sampling 51% of known infections during the same period, representative of different patient categories, we were able to estimate the prevalence of TDR in the country with reasonable accuracy.

The prevalence of TDR in Iceland was found to be at a moderate level (8.5%). This result is slightly higher compared to other Nordic countries.[22,23] Nevertheless; our prevalence estimate was within the range reported in recent studies which investigated the prevalence of TDR in other Western European countries.[24–26] The inclusion of patients diagnosed in the 1990s might have influenced our prevalence estimate. As it is known that the increased ART options together with refined knowledge and improved surveillance have resulted in decreasing levels of resistance,[27] the prevalence of TDR was significantly lower among patients who were diagnosed in the latter half of the study period.

The results of our study have also shown an evidence of decreasing TDR prevalence in Iceland during 1996–2012. This can be attributed to the continued improvements in treatment strategies that successfully suppress viral load and contribute to lower likelihood of transmission.[4] As we have considered other potential explanations for this observation, selection bias seemed to be less likely based on sampling coverage in relation to time and the inclusion of different risk groups and demographic categories. Also the effect of varying proportions of non-B subtypes/CRFs appeared to be marginal, since we found no significant change in proportions between B and non-B subtypes/CRFs over the study period. From 2000 and onwards, the prevalence of TDR appeared to be stabilizing similar to the results reported in other European countries.[24–26,28,29]

Despite lacking statistical significance, the finding that TDR was more prevalent among patients who were born in Iceland and reported infection in the country might be the consequence of the fact that access to widespread ART was established early in Iceland, similar to other European and North American countries. The same reason could explain the higher prevalence of TDR among patients infected with subtype B compared to other HIV-1 genetic variants that dominate in countries where widespread ART was introduced later in the course of HIV-1 epidemic.[30,31]

An interesting finding of our study was that a majority of thymidine analogue 215 revertant mutants circulated among MSM and HET in Iceland between 1997 and 2008 with an estimated median time to the most recent common ancestor of this cluster dating back to 1989. The domestic nature of the TDR cluster was confirmed previously using phylogenetic analysis with similar reference GenBank sequences obtained through BLAST.[3] The likely explanation for the spread of such mutants is the suboptimal AZT-based therapy during late 1980s and early 1990s, in contrast to the later shift towards tenofovir-based combination regimens.[32,33] This observation of domestic spread of TDR highlights the need for continued surveillance to guide the management of HIV-1 infected patients and to limit the spread of such mutant variants. Previous reports in other European countries described the same mutation (T215C/D) circulating domestically.[34,35] The ability of these mutants to be established in transmission chains might be ascribed to their weak effect on the replicative fitness of the virus.[36,37]

### Study limitations

Since the accurate times of infection in all patients were unknown, we considered the Icelandic samples to represent a sero-prevalent cohort. This is an inevitable caveat in most clinical settings since patients are diagnosed at various stages of infection. Another limitation is that the HIV-1 sequences analysed were generated based on the Sanger population sequencing method, and thus minor virus populations might have escaped detection.[38]

## Conclusions

In conclusion, we conducted our investigation on the basis of WHO recommendations for ARV drug resistance surveillance. These results can be valuable in contributing to successful management of HIV-1 infected patients. The results of our study also give useful insights about the effectiveness of the past measures of control and management of these infections. Our findings highlight the importance of continuously monitoring the emergence of ARV resistant mutants in a systematic, prospective manner, to prevent or limit the spread of such mutants which can hinder effective management of HIV-1. Thus, routine baseline HIV-1 genotypic resistance testing in newly diagnosed HIV-infected individuals in the country is recommended to disclose TDR at an early stage which can help to optimize the management of the patients.

## Supplementary Material

Supplementary File
