# Supplementary material for: Decreasing prevalence of transmitted drug resistance among ART-naive HIV-1-infected patients in Iceland, 1996–2012
Source: Infect Ecol Epidemiol. 2017 Jun 13;7(1):1328964. doi: 10.1080/20008686.2017.1328964 (PMC5475329; doi:10.1080/20008686.2017.1328964)
Supplement: Supplementary File [file ziee_a_1328964_sm2096.docx]

**Supplementary File 1: xml file used to conduct Bayesian analysis of 63 Icelandic subtype B sequences**

<?xml version="1.0" standalone="yes"?>

<!-- Supplementary File 1: xml file used to conduct Bayesian analysis of 63 Icelandic subtype B sequences -->

<!-- *Note: All sequences were removed along with omission of all Icelandic sequences identifiers to keep confidentiality of the patients and samples* -->

<!-- Generated by BEAUTi v1.8.4 -->

<!-- by Alexei J. Drummond, Andrew Rambaut and Marc A. Suchard -->

<!-- Department of Computer Science, University of Auckland and -->

<!-- Institute of Evolutionary Biology, University of Edinburgh -->

<!-- David Geffen School of Medicine, University of California, Los Angeles-->

<!-- http://beast.bio.ed.ac.uk/ -->

<beast>

<!-- The list of taxa to be analysed (can also include dates/ages). -->

<!-- ntax=63 -->

<taxa id="taxa">

</taxa>

<taxa id="TDR_1">

</taxa>

<!-- The sequence alignment (each sequence refers to a taxon above). -->

<!-- ntax=63 nchar=1020 -->

<alignment id="alignment" dataType="nucleotide">

</alignment>

<!-- The unique patterns from 1 to end -->

<!-- npatterns=348 -->

<patterns id="patterns" from="1" strip="false">

<alignment idref="alignment"/>

</patterns>

<!-- A prior assumption that the population size has remained constant -->

<!-- throughout the time spanned by the genealogy. -->

<constantSize id="constant" units="years">

<populationSize>

<parameter id="constant.popSize" value="1.0" lower="0.0"/>

</populationSize>

</constantSize>

<!-- Generate a random starting tree under the coalescent process -->

<coalescentSimulator id="startingTree">

<taxa idref="taxa"/>

<constantSize idref="constant"/>

</coalescentSimulator>

<!-- Generate a tree model -->

<treeModel id="treeModel">

<coalescentTree idref="startingTree"/>

<rootHeight>

<parameter id="treeModel.rootHeight"/>

</rootHeight>

<nodeHeights internalNodes="true">

<parameter id="treeModel.internalNodeHeights"/>

</nodeHeights>

<nodeHeights internalNodes="true" rootNode="true">

<parameter id="treeModel.allInternalNodeHeights"/>

</nodeHeights>

</treeModel>

<!-- Taxon Sets -->

<tmrcaStatistic id="tmrca(TDR_1)" includeStem="false">

<mrca>

<taxa idref="TDR_1"/>

</mrca>

<treeModel idref="treeModel"/>

</tmrcaStatistic>

<!-- Generate a coalescent likelihood -->

<coalescentLikelihood id="coalescent">

<model>

<constantSize idref="constant"/>

</model>

<populationTree>

<treeModel idref="treeModel"/>

</populationTree>

</coalescentLikelihood>

<!-- The uncorrelated relaxed clock (Drummond, Ho, Phillips & Rambaut (2006) PLoS Biology 4, e88 )-->

<discretizedBranchRates id="branchRates">

<treeModel idref="treeModel"/>

<distribution>

<logNormalDistributionModel meanInRealSpace="true">

<mean>

<parameter id="ucld.mean" value="0.001" lower="0.0"/>

</mean>

<stdev>

<parameter id="ucld.stdev" value="0.3333333333333333" lower="0.0"/>

</stdev>

</logNormalDistributionModel>

</distribution>

<rateCategories>

<parameter id="branchRates.categories"/>

</rateCategories>

</discretizedBranchRates>

<rateStatistic id="meanRate" name="meanRate" mode="mean" internal="true" external="true">

<treeModel idref="treeModel"/>

<discretizedBranchRates idref="branchRates"/>

</rateStatistic>

<rateStatistic id="coefficientOfVariation" name="coefficientOfVariation" mode="coefficientOfVariation" internal="true" external="true">

<treeModel idref="treeModel"/>

<discretizedBranchRates idref="branchRates"/>

</rateStatistic>

<rateCovarianceStatistic id="covariance" name="covariance">

<treeModel idref="treeModel"/>

<discretizedBranchRates idref="branchRates"/>

</rateCovarianceStatistic>

<!-- The HKY substitution model (Hasegawa, Kishino & Yano, 1985) -->

<HKYModel id="hky">

<frequencies>

<frequencyModel dataType="nucleotide">

<alignment idref="alignment"/>

<frequencies>

<parameter id="frequencies" dimension="4"/>

</frequencies>

</frequencyModel>

</frequencies>

<kappa>

<parameter id="kappa" value="2.0" lower="0.0"/>

</kappa>

</HKYModel>

<!-- site model -->

<siteModel id="siteModel">

<substitutionModel>

<HKYModel idref="hky"/>

</substitutionModel>

<relativeRate>

<parameter id="mu" value="1.0" lower="0.0"/>

</relativeRate>

</siteModel>

<!-- Likelihood for tree given sequence data -->

<treeLikelihood id="treeLikelihood" useAmbiguities="false">

<patterns idref="patterns"/>

<treeModel idref="treeModel"/>

<siteModel idref="siteModel"/>

<discretizedBranchRates idref="branchRates"/>

</treeLikelihood>

<!-- Define operators -->

<operators id="operators" optimizationSchedule="default">

<scaleOperator scaleFactor="0.75" weight="1">

<parameter idref="kappa"/>

</scaleOperator>

<scaleOperator scaleFactor="0.75" weight="3">

<parameter idref="ucld.mean"/>

</scaleOperator>

<scaleOperator scaleFactor="0.75" weight="3">

<parameter idref="ucld.stdev"/>

</scaleOperator>

<subtreeSlide size="1.0" gaussian="true" weight="15">

<treeModel idref="treeModel"/>

</subtreeSlide>

<narrowExchange weight="15">

<treeModel idref="treeModel"/>

</narrowExchange>

<wideExchange weight="3">

<treeModel idref="treeModel"/>

</wideExchange>

<wilsonBalding weight="3">

<treeModel idref="treeModel"/>

</wilsonBalding>

<scaleOperator scaleFactor="0.75" weight="3">

<parameter idref="treeModel.rootHeight"/>

</scaleOperator>

<uniformOperator weight="30">

<parameter idref="treeModel.internalNodeHeights"/>

</uniformOperator>

<scaleOperator scaleFactor="0.75" weight="3">

<parameter idref="constant.popSize"/>

</scaleOperator>

<upDownOperator scaleFactor="0.75" weight="3">

<up>

<parameter idref="ucld.mean"/>

</up>

<down>

<parameter idref="treeModel.allInternalNodeHeights"/>

</down>

</upDownOperator>

<swapOperator size="1" weight="10" autoOptimize="false">

<parameter idref="branchRates.categories"/>

</swapOperator>

<uniformIntegerOperator weight="10">

<parameter idref="branchRates.categories"/>

</uniformIntegerOperator>

</operators>

<!-- Define MCMC -->

<mcmc id="mcmc" chainLength="100000000" autoOptimize="true" operatorAnalysis="B_63.ops.txt">

<posterior id="posterior">

<prior id="prior">

<logNormalPrior mean="1.0" stdev="1.25" offset="0.0" meanInRealSpace="false">

<parameter idref="kappa"/>

</logNormalPrior>

<exponentialPrior mean="0.3333333333333333" offset="0.0">

<parameter idref="ucld.stdev"/>

</exponentialPrior>

<uniformPrior lower="0.0" upper="1.0E100">

<parameter idref="ucld.mean"/>

</uniformPrior>

<oneOnXPrior>

<parameter idref="constant.popSize"/>

</oneOnXPrior>

<coalescentLikelihood idref="coalescent"/>

<discretizedBranchRates idref="branchRates"/>

</prior>

<likelihood id="likelihood">

<treeLikelihood idref="treeLikelihood"/>

</likelihood>

</posterior>

<operators idref="operators"/>

<!-- write log to screen -->

<log id="screenLog" logEvery="10000">

<column label="Posterior" dp="4" width="12">

<posterior idref="posterior"/>

</column>

<column label="Prior" dp="4" width="12">

<prior idref="prior"/>

</column>

<column label="Likelihood" dp="4" width="12">

<likelihood idref="likelihood"/>

</column>

<column label="rootHeight" sf="6" width="12">

<parameter idref="treeModel.rootHeight"/>

</column>

<column label="ucld.mean" sf="6" width="12">

<parameter idref="ucld.mean"/>

</column>

</log>

<!-- write log to file -->

<log id="fileLog" logEvery="10000" fileName="B_63.log.txt" overwrite="false">

<posterior idref="posterior"/>

<prior idref="prior"/>

<likelihood idref="likelihood"/>

<parameter idref="treeModel.rootHeight"/>

<tmrcaStatistic idref="tmrca(TDR_1)"/>

<parameter idref="constant.popSize"/>

<parameter idref="kappa"/>

<parameter idref="ucld.mean"/>

<parameter idref="ucld.stdev"/>

<rateStatistic idref="meanRate"/>

<rateStatistic idref="coefficientOfVariation"/>

<rateCovarianceStatistic idref="covariance"/>

<treeLikelihood idref="treeLikelihood"/>

<discretizedBranchRates idref="branchRates"/>

<coalescentLikelihood idref="coalescent"/>

</log>

<!-- write tree log to file -->

<logTree id="treeFileLog" logEvery="10000" nexusFormat="true" fileName="B_63.trees.txt" sortTranslationTable="true">

<treeModel idref="treeModel"/>

<trait name="rate" tag="rate">

<discretizedBranchRates idref="branchRates"/>

</trait>

<posterior idref="posterior"/>

</logTree>

</mcmc>

<report>

<property name="timer">

<mcmc idref="mcmc"/>

</property>

</report>

</beast>
